# Treatment of Acute Myeloid Leukemia in the Era of Genomics—Achievements and Persisting Challenges

**DOI:** 10.3389/fgene.2020.00480

**Published:** 2020-05-27

**Authors:** Steven D. Green, Heiko Konig

**Affiliations:** Melvin and Bren Simon Comprehensive Cancer Center, Indiana University, Indianapolis, IN, United States

**Keywords:** acute myeloid leukemia, targeted therapies, drug resistance, novel targets, clinical trials

## Abstract

Acute myeloid leukemia (AML) represents a malignant disorder of the hematopoietic system that is mainly characterized by rapid proliferation, dysregulated apoptosis, and impaired differentiation of leukemic blasts. For several decades, the diagnostic approach in AML was largely based on histologic characteristics with little impact on the treatment decision-making process. This perspective has drastically changed within the past years due to the advent of novel molecular technologies, such as whole genome next-generation sequencing (NGS), and the resulting knowledge gain in AML biology and pathogenesis. After more than four decades of intensive chemotherapy as a “one-size-fits-all” concept, several targeted agents have recently been approved for the treatment of AML, either as single agents or as part of combined treatment regimens. Several other compounds, directed against regulators of apoptotic, epigenetic, or microenvironmental pathways, as well as modulators of the immune system, are currently in development and being investigated in clinical trials. The constant progress in AML research has started to produce improved survival rates and fueled hopes that a once rapidly fatal disease can be transformed into a chronic condition. In this review, the authors provide a summary of recent advances in the development of targeted AML therapies and discuss persistent challenges.

## Introduction

Acute myeloid leukemia (AML) is a malignant disorder of the hematopoietic system that typically presents in elderly patients but can occur at any age (American Cancer Society, 2019). With an estimated 5-year overall survival of less than 30%, AML not only represents one of the most fatal leukemias but also ranks among the deadliest of all cancers (National Cancer Institute, 2017). This poor outcome is at least in part due to a myriad of chromosomal alterations and gene mutations that are frequently found in leukemic blasts, thereby promoting a clinically heterogeneous group of diseases which share the common features of high relapse and drug resistance rates (Dohner et al., 2015). Despite steady progress in understanding AML biology and novel technologies to better characterize the disease from an immune-phenotypic and molecular genetic aspect [such as multicolor flow cytometry, droplet digital polymerase chain reaction (ddPCR), and next-generation sequencing (NGS)], treatment concepts have not drastically changed since the 1970s (Lichtman, 2013; Parkin et al., 2017; Shumilov et al., 2018). As such, induction chemotherapy with cytarabine combined with an anthracycline to debulk the initial leukemic burden, followed by two to four cycles of consolidation chemotherapy or an allogeneic stem cell transplant (allo-SCT) to eliminate residual leukemic cells/minimal residual disease (MRD), remain the standard of care for most AML patients today (Tallman et al., 2019). Several attempts to intensify and optimize this chemotherapeutic approach were largely disappointing and thus failed to change the standard of care (Preisler et al., 1987; Dillman et al., 1991). The recognition of the molecular landscape in AML and its impact on vital signaling pathways, such as apoptosis, proliferation, and differentiation, have sparked a new wave of investigational therapies that increasingly utilize targeted agents, either as single agents or combined with standard chemotherapeutic agents, to more efficiently eliminate leukemic cells and improve outcomes. These novel treatment concepts successfully expanded the therapeutic armamentarium and inaugurated a new era in the management of AML. After almost 40 years of little progress, eight new drugs have been approved for AML therapy by the US Food and Drug Administration (FDA) since 2017 (Tiong and Wei, 2019). In this review, we highlight recent achievements in the management of AML patients with a focus on FDA-approved agents, promising drugs in development, and persistent challenges.

## Molecular Targets With FDA-Approved Drugs

### FMS-Like Tyrosine Kinase 3

FMS-like tyrosine kinase 3 (FLT3) gene mutations are present in almost one third of all AML cases. Within this subgroup, internal tandem duplications (ITDs) are most commonly observed. FLT3/ITD-mutated patients typically present with rapidly proliferative disease, high relapse, and short survival rates. FLT3/ITD-mutated AML thus carries a poor prognosis and is placed in the intermediate to adverse risk category per European Leukemia Net (ELN)/National Comprehensive Cancer Network (NCCN) risk stratification (Dohner et al., 2017; Tallman et al., 2019). On April 28, 2017, the FDA approved midostaurin, a small-molecule multi-targeted kinase inhibitor with activity against both FLT3/ITD and FLT3/tyrosine kinase domain (TKD) mutants, for newly diagnosed FLT3-mutated AML patients in combination with chemotherapy. The approval was based on the results of the CALGB10603 (“RATIFY”) study (Stone et al., 2017). In this global, randomized, placebo-controlled Phase III trial of 717 patients with FLT3-mutated AML, the addition of midostaurin to induction and consolidation chemotherapy, followed by 12-month midostaurin maintenance therapy, resulted in superior survival rates compared to chemotherapy alone [7.2% difference in 4-year overall survival (OS)]. Along with the approval of midostaurin, the FDA approved a PCR-based companion diagnostic tool to assess FLT3 mutation status in AML patients. The LeukoStrat CDx mutation assay (Invivoscribe Technologies) is able to reliably detect ITD and TKD mutations with high sensitivity and specificity, and a turnaround time of 2–3 business days (Stone et al., 2017). On November 28, 2018, the agency approved a second FLT3 inhibitor, gilteritinib, for FLT3-mutated, relapsed/refractory disease. In accordance with the final results of the Phase III ADMIRAL study, single-agent gilteritinib significantly improved survival and complete response (CR)/CR with incomplete platelet recovery (CR_P_) rates in FLT3-mutated AML patients compared to salvage chemotherapy [median OS: 9.3 months (gilteritinib) versus 5.6 months (chemotherapy), CR/CR_P_: 34% (gilteritinib) versus 15% (chemotherapy)] (Perl et al., 2019). Several additional FLT3 inhibitors, including a monoclonal anti-FLT3 antibody (LY3012218), are currently in clinical development (Randhawa et al., 2014; Uy et al., 2017a). Of note, FLT3 mutations have also been reported in a variety of solid tumors, such as adenocarcinoma of the lungs, gastrointestinal (GI) cancers, and others (AACR, 2017; Lim et al., 2017). In these settings, reports of clinical activity of FLT3-targeted approaches have been documented, and several clinical trials are currently ongoing (e.g., NCT03329950).

### Isocitrate Dehydrogenase

Somatic mutations within the conserved active site of isocitrate dehydrogenase (IDH) are frequently observed in a wide spectrum of human cancers. In AML, it is estimated that mutations in IDH1 and IDH2 occur in approximately 6–10% and 9–13% of cases, respectively. Physiologically, IDH enzymes catalyze the conversion of isocitrate to α-ketoglutarate (α-KG) in the tricarboxylic acid cycle. However, mutant IDH1 and IDH2 reduce α-KG to the oncometabolite *R*-2-hydroxyglutarate which competitively inhibits α-ketoglutarate-dependent enzymes, leading to epigenetic alterations and impaired hematopoietic differentiation. Ivosidenib (AG-120) and enasidenib (AG-221) are oral, targeted, small-molecule inhibitors of mutant IDH1 and mutant IDH2. On August 01, 2017, the FDA approved enasidenib for adults with relapsed/refractory (R/R) IDH2-mutated AML. The approval was based on the results of study AG221-C-001, an open-label, single-arm, multicenter, two-cohort clinical trial of 199 adult patients with IDH2-mutated R/R AML. Specifically, in this trial, enasidenib demonstrated a combined CR or CR with partial hematologic recovery (CR/CRh) rate of 23% and a median duration of response of 8.2 months (Stein E.M. et al., 2017). Less than 2 years later, on May 02, 2019, ivosidenib was approved for newly diagnosed IDH1-mutated AML patients age 75 years or older or who are deemed unfit for intensive chemotherapy. The approval of the IDH1 inhibitor was based on CR/CRh rates of 42.9% observed in a Phase I dose escalation/expansion study of 258 patients, including 34 patients with untreated AML (Roboz et al., 2018). In conjunction with the IDH inhibitor approvals, the FDA also approved a real-time PCR test to qualitatively detect the presence of five and nine specific mutations in the IDH1 and IDH2 genes, respectively, in the blood or bone marrow (BM) of patients with AML (Dash et al., 2019). The Abbott RealTime assays showed high sensitivity and specificity compared to Sanger sequencing and were able to detect mutations at the 1% level. Similar to the LeukoStrat CDx mutation test for FLT3-mutated patients, the turnaround for this companion diagnostic tool has been reported to be in the range of 3 business days. Outside of AML, IDH mutations have been reported in several solid cancers, such as cholangiocarcinoma, chondrosarcoma, and glioma (Amary et al., 2011; Farshidfar et al., 2017; Huang et al., 2019). IDH-targeted approaches have shown promising activity so far, and multiple clinical trials are underway (Lowery et al., 2019; Nakagawa et al., 2019).

### B-Cell Lymphoma 2

B-cell lymphoma 2 is a gene that encodes an outer mitochondrial membrane protein critical for cell death regulation, including apoptosis, necrosis, and autophagy. Due to its key regulatory function in cellular survival, BCL-2 has represented a promising therapeutic target in various malignancies, including AML, for nearly two decades (Marcucci et al., 2003). After showing promising results as a single agent in clinical trials of lymphoid malignancies, the selective BCL-2 inhibitor venetoclax also demonstrated strong activity in AML (Lin et al., 2016; Stilgenbauer et al., 2016; Wei et al., 2016, 2017). For example, study M14-358, a non-randomized open-label trial combining venetoclax with either decitabine or azacitidine, showed CR rates and a median observed time in remission of up to 54% and 5.5 months, respectively. Encouraging results were also reported in a similar study (M14-387), where venetoclax was combined with low-dose cytarabine (LDAC). In this study, 21% of patients achieved a CR in the setting of a median observed time in remission of 6 months. As a result, on November 21, 2018, the FDA granted accelerated approval to venetoclax in combination with the aforementioned drugs in AML patients who are 75 years or older or unfit to receive standard chemotherapy.

### Hedgehog Signaling Pathway

Lines of evidence suggest that the expression of hedgehog (Hh) signaling proteins such as Smoothened (SMO) and Glioma-Associated Oncogene Homolog 1 (GLI1) is related to treatment resistance in myeloid cells, thus making it a suitable therapeutic target (Zahreddine et al., 2014; Wellbrock et al., 2015; Li et al., 2016). On November 21, 2018, the FDA approved the Hh pathway inhibitor glasdegib in combination with LDAC for the treatment of AML in patients at the age of 75 or older or who are ineligible for intensive chemotherapy. The clinical trial leading to approval was a Phase II study in over 100 patients who were randomized to receive LDAC alone or combined with glasdegib. Patients enrolled on the experimental treatment demonstrated a superior overall survival of 8.3 months compared to 4.3 months for patients treated with LDAC (Cortes et al., 2019).

### CD33

CD33 is a glycosylated transmembrane cell adhesion and interaction molecule whose expression appears to be limited to myeloid cells. Leukemic blasts derived from AML patients express the CD33 surface antigen in up to 90% of cases with high density. On September 01, 2017, the FDA approved the CD33-targeted monoclonal antibody gemtuzumab ozogamicin (GO) for the treatment of newly diagnosed CD33-positive AML in adults and for treatment of relapsed/refractory CD33-positive AML in adults and pediatric patients 2 years and older. The approval was a result of the ALFA-0701 study which included 271 patients with newly diagnosed AML and showed that fractionated doses of GO combined with standard chemotherapy (SC) led to significant improvements in event-free survival (EFS) [13.6 months (GO + SC) versus 8.8 months (SC alone), hazard ratio (HR) 0.68; 95% CI: 0.51–0.91] (Lambert et al., 2019).

## Novel Targets in Development

### Mouse Double Minute 2

Mouse double minute 2 (MDM2) homolog serves as a negative regulator of p53 by both obstructing its DNA binding terminus and by catalyzing its ubiquitination and degradation (Momand et al., 1992; Honda et al., 1997). Due to its potential as a therapeutic target, several inhibitors of MDM2 have been developed to promote wild-type (WT) p53 stabilization and activation. To this end, one group of compounds (so-called nutlins) has been designed to occupy the p53-binding pocket of MDM2. A Phase I/IB study of idasanutlin (RG7388; RO5503781) in combination with cytarabine in 76 patients with R/R AML or those not considered candidates for standard induction therapies reported a composite complete remission (CRc) rate of 29% (22/75) (Giovanni et al., 2016). Preliminary results from a Phase IB study of idasanutlin combined with venetoclax in R/R AML showed clinical activity with an overall response rate (ORR) of 20% and acceptable toxicity (Daver et al., 2017; Table 1). A large, international Phase III study (MIRROS, NCT02545283; Table 1) of idasanutlin combined with cytarabine compared to cytarabine alone in R/R disease is currently ongoing. Other ongoing trials are evaluating idasanutlin as part of induction with cytarabine and daunorubicin, in combination with cytarabine for consolidation, and as single-agent maintenance (NCT03850535; Table 1). Finally, a Phase I study of a pegylated prodrug formulation of idasanutlin (RG7775; RO6839921) as monotherapy in R/R AML reported a CR rate of 7.7% (2/26) and an acceptable safety profile (Yee et al., 2018). Other nutlin compounds are also being developed, such as RG7112 (RO5045337). A Phase I trial enrolling R/R AML patients who were not candidates for standard therapies achieved a CR rate of 10% but at the cost of higher-grade adverse events (AEs), including significant GI toxicity (Andreeff et al., 2016). Another Phase IB trial of this agent in the same patient population but in combination with cytarabine reported encouraging response rates [odds ratio (ORR) rate 43–52%, CR rate 17–21%] (Martinelli et al., 2013). Additional MDM2 inhibitors are in the early phases of human study. Because preclinical studies have suggested that MDM2 inhibition may synergize with mitogen-activated protein kinase kinase (MEK) inhibition, a Phase IB clinical trial of the MDM2 inhibitor AMG 232 as monotherapy or with trametinib in R/R disease has been performed (Erba et al., 2019). In this study, however, dose escalation had to be halted due to GI-related AEs at higher doses. Clinical activity was observed with one CR and one partial response (PR) in the combination arm, and four morphologic leukemia-free states (MLFSs) in the monotherapy arm, out of 30 evaluable patients. Another Phase IB trial in combination with decitabine is currently recruiting (NCT03041688; Table 1). A Phase I trial of the compound HDM201 in patients with R/R AML reported an ORR of 20.6% in 34 patients, no GI dose-limiting toxicities (DLTs), and grade 3/4 effects of cytopenias, tumor lysis syndrome (TLS), and neutropenic fever in >25% (Stein E. et al., 2017). Additional trials testing HDM201 in combination with chemotherapy, or with MBG453 or venetoclax, are recruiting (NCT03760445 and NCT03940352; Table 1). There are four ongoing trials investigating the utility of the oral MDM2 inhibitor milademetan (DS-3032b) administered as monotherapy or combined with LDAC, azacitidine, or quizartinib in unfit or R/R patients (NCT03671564, NCT03634228, NCT02319369, and NCT03552029; Table 1). Of note, the first of a new class of agents known as stapled peptides has been brought to clinical testing with ALRN-6924. It functions as a dual inhibitor of MDM2 and MDM4, another negative regulator of p53, with the goal of abrogating the upregulation of MDM4 that can occur during MDM2 inhibition (Shvarts et al., 1996). A preliminary abstract of a Phase I trial of the agent alone and in combination with cytarabine in R/R *TP53* WT AML reported clinical activity with a manageable side effect profile (Sallman et al., 2018; Table 1).

**TABLE 1:** Ongoing clinical trials of targeted agents.

Target	Drug	Trial phase	Patient population	Single agent/combination	Preliminary results (if available)	Ref/identifier	Status
MDM2	Idasanutlin (RG7388; RO5503781)	IB/II	60+ years old with R/R or secondary AML not eligible for cytotoxic therapy	Combination with venetoclax		NCT02670044	Recruiting
		III	R/R AML	Combination with cytarabine		NCT02545283	Recruiting
		IB/II	Newly diagnosed AML	Combination with cytarabine and daunorubicin for induction; combination with cytarabine for consolidation; single agent for maintenance of CR1		NCT03850535	Recruiting
	AMG 232	IB	R/R or newly diagnosed AML with WT TP53	Combination with decitabine		NCT03041688	Recruiting
	HDM201	I/II	R/R or newly diagnosed AML	Combination with cytarabine, anthracyclines, midostaurin		NCT03760445	Recruiting
		IB	R/R or newly diagnosed AML unfit for standard induction with WT TP53, or high-risk MDS who have failed HMAs	Combination with MBG453 or venetoclax		NCT03940352	Recruiting
	Milademetan (DS-3032b)	I	R/R AML	Monotherapy		NCT03671564	Active, not recruiting
		I/II	R/R AML, WT TP53	Combination with low-dose cytarabine		NCT03634228	Recruiting
		I	R/R AML with WT TP53, or high-risk MDS	Alone and in combination with azacitidine		NCT02319369	Recruiting
		I	R/R or newly diagnosed AML unfit for standard induction with FLT3-ITD mutation	Combination with quizartinib		NCT03552029	Recruiting
	ALRN-6924	I	R/R AML with WT TP53	Alone and in combination with cytarabine	18 patients; no DLTs, only grade 3+ treatment-related AE was thrombocytopenia; 1 patient in combination arm with >50% reduction in blasts	NCT02909972 (Lambert et al., 2019)	Active, not recruiting
p53	APR-246	IB/II	AML with WBC <20K, MDS, MDS/MPN, CMML, with TP53 mutation	Combination with azacitidine		NCT03588078	Active, not recruiting
		II	AML or MDS with TP53 mutation	Combination with azacitidine as maintenance following allo SCT and achievement of CR		NCT03931291	Recruiting
		IB/II	AML (20–30% blasts), MDS, MDS/MPN, CMML, with TP53 mutation	Combination with azacitidine		NCT03072043	Active, not recruiting
	Selinexor (KPT-330)	II	Newly diagnosed AML, 60 years and older	Combination with cytarabine and daunorubicin for induction, with cytarabine for consolidation, and monotherapy for maintenance		NCT02835222	Recruiting
		IB	R/R AML or DLBCL	Combination with venetoclax		NCT03955783	Recruiting
		I	AML and high-risk MDS after allo-SCT	Monotherapy to eliminate MRD and maintain remission		NCT02485535	Active, not recruiting
		I	R/R AML	Combination with FLAG-Ida		NCT03661515	Recruiting
	Arsenic trioxide	II	AML with TP53 mutation	Combination with decitabine and cytarabine		NCT03381781	Not yet recruiting
Aurora kinase	AZD2811	I/II	Newly diagnosed AML not eligible for intensive induction, or R/R AML	Alone or in combination with azacitidine		NCT03217838	Recruiting
PLK	Onvansertib (PCM-075)	I/II	R/R AML	Combination with LDAC or decitabine	21 patients. No serious treatment-related AEs at the first 3 doses. Responses in 5 of 19 evaluable pts (2 CRc, 1 PR, and 2 MLFS). Greatest response in the onvansertib and decitabine arm with 2/4 patients achieving CRc.	NCT03303339 (Dennis et al., 2012)	Recruiting
	CFI-400945	I	R/R AML or MDS	Monotherapy		NCT03187288	Recruiting
DOT1L	Pinometostat (EPZ-5676)	IB/II	Newly diagnosed AML with MLL rearrangement	Combination with cytarabine and daunorubicin induction		NCT03724084	Recruiting
		IB/II	Newly diagnosed or R/R AML with MLL rearrangement	Combination with azacitidine		NCT03701295	Recruiting
IL-3 receptor alpha (CD123)	SL-401	I/II	AML in CR; any MRD status for stage 1 and MRD+ for stage 2	Monotherapy	9 patients in Stage 1 and 7 patients in Stage 2. No DLTs; most common grade 3+ treatment-related AEs were transaminitis and thrombocytopenia; two with grade 3 capillary leak syndrome. MRD and DFS analysis for Stage 2 is ongoing	NCT02270463 (Dohner et al., 2014b)	Active, not recruiting
		I/II	Newly diagnosed treatment-related or secondary AML, R/R AML, and blastic plasmacytoid dendritic cell neoplasm (BPDCN)	Monotherapy	Results published for BPDCN but not AML	NCT02113982	Active, not recruiting
		I	Newly diagnosed AML not eligible for intensive induction, R/R AML, High-risk MDS. CD123 expression confirmed	Combination with azacitidine		NCT03113643	Recruiting
Bromodomain	Mivebresib (ABBV-075)	I	R/R AML, as well as other solid or hematologic malignancies that are refractory or for which standard of care does not exist	Alone and in combination with venetoclax	41 patients (19 monotherapy and 22 combination). No DLTs; common grade 3+ AEs of cytopenias. Only efficacy data reported so far is median best percentage BM blast change for 26 evaluable pts which was −20% (range −98% to +300%)	NCT02391480 (Nguyen et al., 2011)	Completed
	GSK525762	I/II	R/R AML or those older than 65 not candidates for/refused standard chemotherapy	Monotherapy	46 pts. DLTs included diarrhea and decreased EF. Common grade 3+ AEs were diarrhea, febrile neutropenia, thrombocytopenia, hyperglycemia, and fatigue. 2 CRcs and 3 PRs. Most responses were delayed and required at least 10 weeks of therapy to manifest	NCT01943851 (Bernt et al., 2011)	Recruiting
	RO6870810/ TEN-010	I	R/R AML and MDS	Monotherapy	Not yet published	NCT02308761	Completed
	FT-1101	I/IB	AML, MDS, NHL that is R/R or for whom standard treatment are contraindicated	Alone and in combination with azacitidine	Not yet published	NCT02543879	Completed
	ABBV-744	I	R/R AML and metastatic castrate-resistant prostate cancer	Monotherapy		NCT03360006	Recruiting
	PLX51107	I	AML and MDS	Combination with azacitidine		NCT04022785	Not yet recruiting
CTLA-4	Ipilimumab	I	Intermediate-II, high-risk, or any FLT3+ AML; IPSS intermediate-2 or high-risk MDS	Allogeneic HSCT followed by ipilimumab, ipilimumab + nivolumab, or nivolumab.		NCT02846376	Recruiting
		I	R/R AML or MDS following allogeneic HSCT	Allogeneic HSCT followed by ipilimumab, ipilimumab + nivolumab, or nivolumab		NCT03600155	Recruiting
		I	R/R AML or newly diagnosed AML 75 years or older; R/R MDS.	Combination with decitabine.		NCT02890329	Recruiting
		II	R/R AML, newly diagnosed AML age 65+ unfit for/decline standard induction, or newly diagnosed secondary AML	Azacitidine and nivolumab ± ipilimumab.		NCT02397720	Recruiting
MCL-1	AMG 176	I	R/R AML or MM	Alone and in combination with azacitidine (AML) and carfilzomib (MM)		NCT02675452	Active, not recruiting
	AMG 397	I	R/R AML, MM, NHL	Monotherapy.		NCT03465540	Active, not recruiting
	S64315	I	R/R AML, or AML secondary to MDS treated at least with HMA, or untreated AML if >65 and not candidate for intensive/alternative chemotherapy; R/R MDS.	Monotherapy		NCT02979366	Recruiting
		IB	R/R AML, or AML secondary to MDS treated at least with HMA and without established alternative therapy, or untreated AML if >65 and not candidate for intensive/alternative chemotherapy	Combination with venetoclax		NCT03672695	Recruiting
	AZD5991	I/II	R/R AML as well as other R/R hematologic malignancies; must have received at least 2 prior lines of therapy	Alone and in combination with venetoclax		NCT03218683	Recruiting
CD47	Magrolimab (Hu5F9-G4; 5F9)	IB	Newly diagnosed AML not eligible for intensive induction, or untreated intermediate to very high risk MDS	Alone and in combination with azacitidine	22 pts with AML. Common treatment-related AEs were cytopenias. No MTD reached. ORR 64% (14/22), CR 41%, CRi 14%, PR 5%, MLFS 5%. MRD negativity noted in 57% of responders	NCT03248479 (Glaser et al., 2012)	Recruiting
		IB	R/R AML	Combination with atezolizumab		NCT03922477	Recruiting
	TTI-621 (SIRPaFc)	IA/IB	R/R hematologic malignancies and selected solid tumors	Monotherapy		NCT02663518	Recruiting

*Allogeneic HSCT, allogeneic hematopoietic stem cell transplantation; AML, acute myeloid leukemia; CR, complete response; MDS, myelodysplastic syndrome; MM, multiple myeloma; NHL, non-Hodgkin lymphoma; ORR, overall response rate; PR, partial response; R/R, relapsed/refractory.*

### p53

The *TP53* gene is located on the short arm of chromosome 17 and encodes the transcription factor and tumor suppressor protein p53, which acts as a barrier to leukemic stem cell formation (Aloni-Grinstein et al., 2014). p53 activation primarily results in increased transcription of p21, which binds to cyclin-dependent kinases (CDKs) inhibiting the *G*_1_/*S* phase transition, and evokes cellular senescence and apoptosis (Harper et al., 1993). Mutations of *TP53* in AML are leukemogenic drivers and are of prognostic importance because they are often associated with drug resistance and poor outcomes (Rucker et al., 2012; Dohner et al., 2017; Tallman et al., 2019). Although rare in *de novo* AML, *TP53* mutations are enriched in secondary and therapy-related AML as well as in cases of cytogenetically complex disease (5). Historically, the field has focused on loss-of-function mutations, but recent discoveries are demonstrating the prevalence of p53 gain-of-function mutations and non-mutational WT p53 dysfunction in AML (Prokocimer et al., 2017). While the true frequency and oncogenicity of these abnormalities remain unknown, the recognition of the variety of mechanisms resulting in aberrant p53 function is yielding new therapeutic targets. Reactivating loss-of-function mutant p53 is being studied in clinical trials with the agent APR-246 (PRIMA-1MET). Its metabolite binds to the core domain of mutant p53, stimulating proper folding and restoring DNA binding, and induces the production of reactive oxygen species (Bykov et al., 2002; Lambert et al., 2009). A first-in-human Phase I trial of APR-246 included seven patients with refractory AML and demonstrated that the drug was well-tolerated with DLTs of increased alanine aminotransferase (ALT)/aspartate aminotransferase (AST), dizziness, confusion, and sensory disturbances and had a favorable pharmacokinetic (PK) profile (Lehmann et al., 2012). There are several ongoing Phase I and II clinical trials in combination with azacitidine in patients with *TP53* mutations (NCT03588078, NCT03931291, and NCT03072043; Table 1). Mutant p53 can also bind to heat shock protein 90 (HSP90), preventing MDM2 binding and degradation *via* ubiquitylation (Li et al., 2011). Therefore, several drugs that inhibit HSP90 have been developed. A Phase I study of cytarabine and the HSP90 inhibitor tanespimycin (17-AAG) in 22 R/R AML patients reported treatment-related AEs of disseminated intravascular coagulation (DIC), acute respiratory distress syndrome (ARDS), and myocardial infarction (MI), and the maximum tolerated dose (MTD) only exposed patients to effective concentrations for a brief time (Kaufmann et al., 2011). Similarly, a Phase I study of tanespimycin in combination with bortezomib in R/R AML enrolling 11 patients regardless of *TP53* mutation status demonstrated toxicity without a measurable response (Walker et al., 2013). The HSP90 inhibitor ganetespib (STA-9090) has also been studied, with Phase I data demonstrating that it is well-tolerated and has a favorable PK profile with preliminary signs of pharmacodynamic activity (Lancet et al., 2010; Padmanabhan et al., 2010). A Phase I/II clinical trial with an arm delivering chemotherapy (daunorubicin, cytarabine, and etoposide) and ganetespib is completed, but results are not yet available (NCT01236144). Another mode of p53 dysfunction in AML that is being therapeutically targeted is overexpression of XPO1/CRM1, resulting in nuclear export of p53. A class of oral small-molecule XPO1 inhibitors known as selective inhibitors of nuclear export (SINEs) redirects wtp53 to the nucleus, thus promoting its transcriptional activities (Senapedis et al., 2014). The most thoroughly studied is KPT-330 (selinexor). A Phase I trial of single-agent selinexor in R/R AML demonstrated no DLTs (the most frequent adverse effects were grade 1/2 constitutional and GI toxicities) and an ORR of 14% (11/81) (Garzon et al., 2017). Other Phase I studies with published results have examined selinexor in R/R patients combined with cladribine plus cytarabine (CLAG), mitoxantrone plus etoposide plus cytarabine (MEC), cytarabine and idarubicin, and sorafenib; and in untreated patients combined with high-dose cytarabine and mitoxantrone, cytarabine and daunorubicin, and decitabine (Bhatnagar et al., 2016; Fiedler et al., 2016; Sweet et al., 2016; Uy et al., 2017b; Bhatnagar et al., 2018; Wang et al., 2018; Zhang et al., 2018). Overall, they demonstrate that the agent is tolerable and feasible in combination approaches. A Phase II study of selinexor plus cytarabine and idarubicin in patients with R/R disease achieved a CR/CR with incomplete hematologic recovery (CRi) in 20 of 42 patients (48%) (Walter et al., 2019). Another Phase II trial (SOPRA) randomized R/R AML patients 60 years and older ineligible for intensive therapy to either selinexor (*n* = 118) or physician’s choice of hydrea, LDAC, or hypomethylating agent (HMA) (*n* = 57). The trial did not meet its primary endpoint, as the median OS was 94 days in the selinexor arm compared to 170 days in the control arm (*p* = 0.4221) (Kendra et al., 2019). Ongoing trials are investigating combination therapy with venetoclax and its potential to eliminate MRD after allo-SCT (NCT02835222, NCT03955783, NCT02485535, and NCT03661515; Table 1). Other approaches being pursued include the p53 antisense oligonucleotide EL625 (Cenersen) and a forthcoming trial incorporating arsenic trioxide based on its preclinical role in regulating p53 (Cortes et al., 2012; NCT03381781; Table 1).

### Aurora Kinase

The serine–threonine Aurora kinases are integral to mitotic processes such as spindle assembly, centrosome separation, and chromosome segregation (Glover et al., 1995). Overexpression has been observed in leukemic cells and is associated with aneuploidy, genetic instability, and phosphorylation of p53 (Katayama et al., 2004; Khan et al., 2011; Yang et al., 2013). Inhibition of Aurora kinases *in vitro* results in aneuploidy and apoptosis (Hoar et al., 2007). Based on this data and early clinical trials in solid cancers, a Phase II study of the oral Aurora A kinase inhibitor alisertib (MLN8237) was conducted (Goldberg et al., 2014). It enrolled 35 patients with AML who were ineligible for intensive induction or had R/R disease. The ORR was 17% (6/35) with one CR and five PRs, and 12 patients achieving transfusion independence. The most common AEs were diarrhea, fatigue, nausea, febrile neutropenia, and stomatitis. A delayed time to first response suggests that multiple cycles may be needed to appreciate its antileukemic activity. Subsequently, a Phase I clinical trial tested the combination of alisertib with induction chemotherapy (cytarabine and idarubicin) in newly diagnosed AML, with the option of incorporating the investigational agent into consolidation and maintenance therapy (Fathi et al., 2017). Twenty-two patients were enrolled, achieving a CRc rate of 86% (19/22), including 7/8 patients over the age of 65 and 10/10 patients with high-risk AML. With a median follow-up of 13.5 months, the 12-month OS and progression-free survival (PFS) for those treated with the recommended Phase II dose was 62% and 42%, respectively. Ultimately, 45% (*n* = 10) received at least one cycle of consolidation, and 18% (*n* = 4) received maintenance with alisertib. A Phase II trial utilizing the same treatment regimen was conducted in newly diagnosed high-risk AML patients (defined as poor-risk cytogenetics, secondary/treatment-related, or age 65+) and achieved a CRc rate of 64% (25/39), meeting its primary endpoint (Brunner et al., 2018). With a median follow-up of 14 months, the median OS was 12.2 months (90% CI: 8.8–NA), although data continue to mature. In those achieving CRc, the 12-month relapse-free survival (RFS) was 52%. This remains the most promising candidate of the Aurora kinase inhibitor class. Selective Aurora B kinase inhibition has also been investigated with barasertib (AZD1152) in Phase I and II studies. A Phase I/II study in R/R or newly diagnosed AML patients not candidates for standard induction enrolled 64 patients (Lowenberg et al., 2011). It noted common grade 3+ AEs of febrile neutropenia and mucositis. The ORR was 26% at the MTD, with a CRc rate of 18% across all prognostic risk groups. Two other small Phase I studies using the same dosing and patient population reported a similar safety profile and ORR of 19–25% (Tsuboi et al., 2011; Dennis et al., 2012). The Phase II SPARK-AML1 trial compared barasertib to LDAC in newly diagnosed patients 60 years and older considered unsuitable for intensive induction (Kantarjian et al., 2013). The study enrolled 74 patients (48 barasertib; 26 LDAC) and met its primary endpoint of objective CR rate (35.4% versus 11.5%; *p* < 0.05) across all cytogenetic risk groups. Although the study was not powered to detect an improvement in OS, with a median follow-up of 12.9 months, there was no significant difference in OS. Grade 3+ AEs were seen in 83% versus 69%. Ultimately, the fact that the drug is administered as a 7-day continuous infusion limited its clinical utility and led to discontinuation of its development. However, a nanoparticle formulation of AZD2811, the active metabolite of barasertib, has recently been shown to have activity in preclinical models and can be administered as an infusion over several hours (Floc’h et al., 2017). Therefore, a Phase I/II clinical trial of AZD2811 nanoparticles as monotherapy or in combination with azacitidine is currently recruiting (NCT03217838; Table 1). Less specific small-molecule inhibitors of the aurora kinases have also been explored; however, Phase I trials of the pan-aurora kinase inhibitors AMG 900 and AT9283 yielded poor response rates as monotherapy and significant toxicity (Foran et al., 2014; Kantarjian et al., 2017). It is unclear how much of these agents’ antileukemic and toxic effects are specifically related to Aurora kinase inhibition versus other targets.

### Polo-Like Kinase

Polo-like kinases (PLKs) are involved in the regulation of mitosis and cellular division (Lens et al., 2010). They have been found to be overexpressed in AML, with inhibition resulting in mitotic arrest and apoptosis (Steegmaier et al., 2007; Renner et al., 2009). Two early-phase trials of the PLK1-3 inhibitor volasertib (BI 6727) in patients ineligible for standard induction or with R/R disease reported ORRs of 12 and 36.8%. Grade 3+ toxicities of cytopenias, mucositis, febrile neutropenia, and pneumonia (PNA), as well as DLTs of infection, mucositis, thrombocytopenia, abnormal LFTs, and bleeding were noted (Bug et al., 2011; Dohner et al., 2014a; Kobayashi et al., 2015). A Phase I/II study of the agent in combination with LDAC demonstrated an ORR of 22% (7/32) (Bug et al., 2010, 2011). This led to the design of a Phase II trial of LDAC with or without volasertib in untreated AML patients not considered suitable for standard induction therapy (Dohner et al., 2014b). Eighty-seven patients were enrolled with a median age of 75 years. The primary endpoint was objective response (CRc), which was higher for the combination arm compared to LDAC alone (31% versus 13.3%) but was not statistically significant (*p* = 0.052). Although not powered for survival endpoints, the median OS was 8.0 months versus 5.2 months (*p* = 0.047). There was an increased frequency of neutropenic fever, infections, and GI events without an increase in death rate at days 30, 60, and 90. Based on these results, the placebo-controlled, double-blind, Phase III POLO-AML-2 trial was conducted (Döhner et al., 2016). This compared the same treatment regimens in the same patient population, with the additional inclusion criteria of age 65 years or older. Patients were randomized 2:1 to combination therapy or LDAC alone, with stratification by Eastern Cooperative Oncology Group (ECOG) status and type of AML (*de novo* versus secondary). The primary endpoint was again CRc, with OS a secondary endpoint. The primary analysis included 246 patients in the combination arm and 125 patients in the LDAC monotherapy group, with a median age of 75 years and an ORR of 25.2% versus 16.8% (*p* = 0.071). Therefore, its primary endpoint was not met, and in fact a negative median OS trend was seen (4.8 months versus 6.5 months; *p* = 0.113). The AE severity was increased in the combination arm, with a fatal infection frequency of 16.6% versus 5.1% considered to be the main reason for the negative OS trend. Other PLK inhibitors currently under investigation include the third-generation oral PLK1 inhibitor onvansertib (PCM-075) and the PLK4 inhibitor CFI-400945 (Zeidan et al., 2019b; NCT03187288; Table 1). A Phase I trial currently recruiting is testing dose escalation of onvansertib in combination with LDAC or decitabine in patients with R/R disease (Zeidan et al., 2019b). Preliminary data published in abstract form reports enrolling 21 patients with no serious treatment-related AEs at the first three doses. Responses have been seen in 5 of 19 evaluable patients (two CRc, one PR, and two MLFS). The greatest response has been in the onvansertib and decitabine arm with 2/4 patients achieving CRc. A Phase I trial of the oral PLK4 inhibitor CFI-400945 is currently recruiting patients with R/R AML or myelodysplastic syndrome (MDS) (NCT03187288). Rigosertib, a non-specific inhibitor of PLK1 as well as phosphoinositide 3-kinase (PI3K) and the Ras pathway, has primarily been studied for the treatment of MDS. However, a Phase I/II study included 13 patients with R/R AML arising from an antecedent MDS and achieved a PR in 1/13. Grade 3+ toxicities of cytopenias, electrolyte disturbances, PNA, and hypoglycemia were reported (Navada et al., 2018).

### Disruptor of Telomeric Silencing 1-Like

Rearrangements of the myeloid/lymphoid or mixed lineage leukemia (MLL) gene (also known as KMT2A) on chromosome 11q23 are seen in approximately 2–10% of AML cases, are enriched in therapy-related AML, and carry a poor prognosis (Schoch et al., 2003; Tallman et al., 2019). When rearranged, the locus generates chimeric proteins that aberrantly recruit disruptor of telomeric silencing 1-like (DOT1L), an H3K79 (histone 3 lysine 79) methyltransferase. DOT1L then methylates and promotes the expression of genes associated with leukemogenesis, including HOXA9 and MEIS1 (Milne et al., 2002; Bernt et al., 2011; Nguyen et al., 2011). DOT1L inhibition was therefore explored in preclinical studies and resulted in reduced expression of leukemogenic genes and leukemic cell death (Daigle et al., 2011). The selective DOT1L small-molecule inhibitor pinometostat (EPZ-5676) has been studied in a Phase I dose escalation and expansion clinical trial (Stein et al., 2018). Forty-three patients with R/R AML were enrolled, 37 of whom had MLL gene rearrangements and/or partial tandem duplications. Expansion-phase participants were restricted to those with MLL abnormalities. There were no DLTs; however, five patients had grade 3+ drug-related cardiac AEs (heart failure, decreased ejection fraction, cardiopulmonary arrest). One patient experienced a CR. A logistically hindering feature of the drug is that it requires continuous intravenous infusion for 28 days because of the sustained exposure required for antitumor activity and rapid decline when stopped. Further preclinical work has demonstrated that pinometostat has increased activity when combined with chemotherapy and hypomethylating agents, leading to two ongoing Phase I/II clinical trials investigating the agent in combination with standard induction chemotherapy or azacitidine in newly diagnosed and R/R AML harboring an MLL rearrangement (Klaus et al., 2014; NCT03724084 and NCT03701295; Table 1).

### CD123

Myeloid leukemic progenitor cells overexpress interleukin (IL)-3 receptor alpha chain (CD123), which binds to IL-3 and undergoes receptor-mediated endocytosis (Jordan et al., 2000). SL-401 (tagraxofusp) is a recombinant fusion protein that leverages this expression by combining IL-3 with a truncated diphtheria toxin that inactivates protein synthesis when endocytosed and is toxic to leukemic blasts (Frankel et al., 2000). A Phase I dose escalation trial enrolling 40 patients with R/R AML or those with untreated disease age 70 and older achieved one CR and one PR (Frankel et al., 2008). Common AEs included transient transaminitis and manifestations of capillary leak such as hypoalbuminemia, edema, and hypotension. A Phase I/II trial enrolling 59 patients with R/R AML administered a single cycle of SL-401 and achieved two CRs with a similar toxicity profile (Frankel et al., 2013). Preliminary results from an ongoing Phase I/II dose escalation and expansion trial of SL-401 as consolidation therapy for patients in remission have been presented (Lane et al., 2017; Table 1). Stage 1 of the trial enrolled patients in first or second remission (CR1 or CR2) regardless of MRD status. Stage 2 patients were those in remission who were MRD+, with the goal of reducing the residual presumptively chemoresistant cell population. An abstract has reported enrolling nine patients in Stage 1 and seven patients in Stage 2, with median baseline MRD in the range of 0.4–4%. MRD and disease-free survival (DFS) analysis for Stage 2 is ongoing. There were no DLTs, and the most common grade 3+ treatment-related AEs were transaminitis and thrombocytopenia. A Phase II study recruited 30 patients with R/R AML as well as patients with blastic plasmacytoid dendritic cell neoplasm; however, the data for AML has not yet been published (NCT02113982; Table 1). Finally, there is an ongoing Phase I study of SL-401 in combination with azacitidine in R/R or treatment-naive AML not eligible for standard induction that is currently recruiting (NCT03113643; Table 1).

### Bromodomains

The bromodomain (BRD) and extraterminal (BET) class of proteins are integral to the epigenetic regulation of gene transcription (Filippakopoulos and Knapp, 2014). They bind to acetylated histone tails and associate with enhancers and positive transcription factor b (P-TEFb) to promote elongation and transcription (Jang et al., 2005). As a result, these proteins often regulate the expression of key oncogenes and antiapoptotic proteins and have become a therapeutic target of interest (Filippakopoulos et al., 2010; Zuber et al., 2011). The first bromodomain inhibitor studied clinically in AML was OTX015 (MK-8628), an oral BRD2-4 inhibitor. A Phase I dose escalation trial enrolled R/R patients <60 years old who had failed at least two standard regimens or older patients who had failed at least one regimen and relapsed within 1 year or had a contraindication for standard therapies (Berthon et al., 2016). Thirty-six patients were recruited with a median age of 70, and two patients achieved a CRc. DLTs of diarrhea and fatigue were noted, as well as common grade 3+ AEs of fatigue and bilirubin elevation. Given its modest efficacy as monotherapy, the authors concluded that the agent will need to be utilized as part of combination therapy or predictive biomarkers will need to be identified. Mivebresib (ABBV-075) is a pan-BET inhibitor currently being tested in a Phase I study as monotherapy or combined with venetoclax in patients with R/R AML. Preliminary data published in abstract form reports enrolling 41 patients with common grade 3+ AEs of febrile neutropenia, anemia, and thrombocytopenia (Odenike et al., 2019; Table 1). The only efficacy data reported have been the median best percentage BM blast change for 26 evaluable patients, which was −20% (range −98% to +300%). The final bromodomain inhibitor with preliminary clinical trial data available at this time is GSK525762, which has been studied in a Phase I trial in patients with R/R disease or those older than 65 years who were not candidates for/refused standard chemotherapy (Dawson et al., 2017; Table 1). Forty-six patients were enrolled with two CRcs and three PRs observed. Most responses were delayed requiring at least 10 weeks of therapy to manifest. DLTs included diarrhea, febrile neutropenia, thrombocytopenia, hyperglycemia, and fatigue. Phase I trials of the compounds RO6870810/TEN-010, FT-1101, ABBV-744, and PLX51107 as monotherapy or in combination with azacitidine are ongoing or have been completed, but results are not yet published (NCT02308761, NCT02543879, NCT03360006, and NCT04022785; Table 1).

### Cytotoxic T-Lymphocyte-Associated Protein 4

Cytotoxic T-lymphocyte-associated protein 4 (CTLA-4) functions as a negative regulator of T-cell activation and effector function and is expressed by myeloid leukemia cells (Pistillo et al., 2003). CTLA-4 inhibition *in vitro* leads to apoptosis of leukemia cells (Laurent et al., 2007). A Phase I/IB study of the CTLA-4 inhibitor ipilimumab administered to patients with hematologic malignancies who relapsed after allo-SCT enrolled 12 patients with AML (Davids et al., 2016). Five of the 12 AML patients responded, including four CRs in patients with extramedullary disease. Immune-related AEs were observed in 21% of patients in the trial, and graft-versus-host disease (GVHD) that precluded further administration of ipilimumab was observed in 14%. There are ongoing clinical trials studying ipilimumab as monotherapy and in combination with nivolumab following allo-SCT in high-risk patients and in patients with R/R disease following transplant (NCT02846376 and NCT03600155; Table 1). Preclinical studies have demonstrated that hypomethylating agents can upregulate CTLA-4 expression (Yang X. et al., 2014). Therefore, there are ongoing clinical trials testing ipilimumab in combination with decitabine, azacitidine, and nivolumab (NCT02890329 and NCT02397720; Table 1).

### Induced Myeloid Leukemia Cell Differentiation Protein MCL-1

Induced myeloid leukemia cell differentiation protein MCL-1 is an antiapoptotic protein in the BCL2 family which sequesters proapoptotic proteins such as Bcl-2-associated X (BAX) and Bcl-2 homologous antagonist killer (BAK) (Willis et al., 2005). Overexpression has been observed in AML and is associated with resistance to therapies such as venetoclax (Tahir et al., 2017). Preclinical studies indicate that MCL-1 inhibition leads to apoptosis of AML cells (Glaser et al., 2012). Selective antagonists of MCL-1 (AMG 176, AMG 397, S64315, and AZD5991) have been developed and are being tested in ongoing early-phase clinical trials as monotherapy and in combination with venetoclax (NCT02675452, NCT03465540, NCT02979366, NCT03672695, and NCT03218683; Table 1). Enrollment in the AMG 397 study is currently on hold after a cardiac toxicity signal was seen, with the AMG 176 trial placed on a voluntary hold as well. There are several other agents such as the cyclin-dependent kinase (CDK) inhibitors that are not specific for MCL-1 but cause downregulation of MCL-1 as part of their mechanism of action (Hird and Tron, 2019). However, it is therefore unclear how much of their therapeutic effect is the result of MCL-1 inhibition. Trials are underway attempting to correlate the efficacy of agents such as the CDK9 inhibitor alvocidib (DSP-2033; flavopiridol) to MCL-1 dependency (NCT03298984 and NCT02520011).

### CD47

CD47 generates an anti-phagocytic signal upon binding its ligand Signal Regulatory Protein Alpha (SIRPa) on macrophages by inhibiting myosin accumulation at the phagocytic synapse (Tsai and Discher, 2008). The discoveries that CD47 is overexpressed on AML cells, that its increased expression is an independent poor prognostic factor, and that inhibitory antibodies can successfully enable phagocytosis of leukemic cells and initiate an antitumor T-cell response, led to the further investigation of CD47 as a therapeutic target (Jaiswal et al., 2009; Majeti et al., 2009; Tseng et al., 2013; Liu et al., 2015). Magrolimab (Hu5F9-G4; 5F9) is an immunoglobulin G (IgG)4 anti-CD47 monoclonal antibody that was well-tolerated in a Phase I study of 15 R/R AML patients in the UK (Vyas et al., 2018). Common treatment-related AEs were anemia, hemagglutination, pyrexia, and headache, but no DLTs were observed. No objective responses were observed either, and therefore it was determined to be inappropriate for further development as monotherapy. However, preclinical studies noted that magrolimab synergizes with azacitidine perhaps by upregulating the pro-phagocytic protein calreticulin (Feng et al., 2018). Therefore, a Phase IB clinical trial tested the combination in untreated AML patients ineligible for induction chemotherapy (Sallman et al., 2019; Table 1). The combination was well-tolerated, with no MTD reached and a safety profile consistent with Azacitidine (AZA) monotherapy. An interesting aspect of the biology of this target is that CD47 appears to be an important homeostatic regulator of red blood cell (RBC) turnover, and therefore blockade can lead to accelerated RBC clearance *via* phagocytosis and resultant anemia (Oldenborg et al., 2000). In this Phase IB trial, an initial priming dose followed by maintenance dosing was successful in mitigating the anemia and resulted in a transient mild hemoglobin (Hgb) drop with subsequent return to baseline. Preliminary efficacy results demonstrate an ORR of 64% (14/22; 41% CR, 14% CRi) including 78% (7/9) of those with TP53 mutations. MRD negativity was noted in 57% of responders. The median time to response was 1.9 months, which is more rapid than AZA alone. Based on these results, the FDA granted magrolimab fast track designation in September 2019. An additional Phase IB study evaluating the combination of magrolimab with the PD-L1 antagonist atezolizumab in patients with R/R AML is currently recruiting (NCT03922477; Table 1). Another agent targeting this pathway is TTI-621 (SIRPaFc), an IgG1 SIRPa-Fc fusion protein that functions as a CD47 antagonist and also contains an Fc region which can interact with Fcg receptors on macrophages to further enhance phagocytosis (Petrova et al., 2017). A Phase IA/IB trial of TTI-621 in patients with R/R hematologic malignancies and select solid tumors is currently ongoing, but the data for the AML cohort have not yet been published (NCT02663518; Table 1). Finally, the anti-CD47 monoclonal antibody CC-90002 was tested in a Phase I study of patients with R/R AML; however, the agent was poorly tolerated, no objective responses were observed, and the development of anti-drug antibodies was noted at all dose levels (Zeidan et al., 2019a). The trial was therefore terminated.

### Histone Deacetylase

As epigenetic dysregulation is known to contribute to the pathogenesis of AML, therapies targeting epigenetic alterations such as acetylation are under clinical development. The acetylation of lysine residues of histone tails results in a weakening of the electrostatic interaction between the histone protein and DNA, allowing for the binding of transcription factors and gene expression (Grant, 2001). Histone deacetylase (HDAC) inhibitors (HDACi) alter the acetylation and therefore regulation of both histone and non-histone proteins, including oncogenes and tumor-suppressor genes (Roy et al., 2005; Peng and Seto, 2011). Despite promising preclinical studies, HDACi do not seem to be effective as monotherapies in AML but are being investigated in combination with other drugs (San José-Enériz et al., 2019). For example, the HDACi belinostat and the NEDD8-activating enzyme pevonedistat (MLN4924) appear to synergistically induce apoptosis of AML cells by disrupting the DNA damage response (Zhou et al., 2016). A Phase I study of the combination in R/R AML patients is currently recruiting (NCT03772925).

## Persistent Challenges

Chemotherapy for the treatment of AML traditionally consists of an induction phase, aimed at debulking the leukemic burden, and a consolidation phase, directed against residual leukemic cells, including leukemic stem cells (LSCs) that remain after initial therapies. It is estimated that in response to induction chemotherapy up to 75% of patients with newly diagnosed AML achieve a morphologic leukemia-free state (complete remission), which constitutes a prerequisite to receive consolidation treatment (Burnett et al., 2015). Given the structural design of classical AML therapy, novel agents need to be tested in each setting in order to assess their ability to debulk the initial burden and/or eradicate residual leukemic cells and LSCs, either as a single agent or when combined with conventional chemotherapy. This approach is of critical importance as, for example, a targeted inhibitor with strong debulking activity might be less effective against residual leukemic cells, and vice versa. Moreover, patients could potentially be spared the toxic side effects of an agent that might confer only limited activity in the early or late phases of therapy. Intriguingly, when tested in newly diagnosed AML, targeted inhibitors are frequently added to standard chemotherapies in both induction and consolidation arms. An example is the incorporation of FLT3 inhibitors into treatment regimens against FLT3-mutated AML. While lines of evidence suggest that FLT3 inhibitors exert their anti-leukemic activity through induction of apoptosis and terminal differentiation of leukemic blasts in the peripheral blood and BM, respectively, one would expect these mechanisms to translate into improved remission and survival rates (Sexauer et al., 2012; Yang H. et al., 2014). In the RATIFY trial, however, the addition of the FLT3 inhibitor midostaurin to both induction and consolidation therapy did not result in improved remission rates [CR 59% (midostaurin group) versus 54% (placebo group), *p* = 0.15], whereas survival rates were significantly superior in the experimental arm (Stone et al., 2017). These results suggest that the clinical benefit of adding an FLT3 inhibitor to standard chemotherapy might mostly result from consolidation treatment and thus the continuous exposure of residual blasts to the inhibitor. Similarly, a study of gemtuzumab ozogamicin (GO) combined with conventional frontline chemotherapy compared to chemotherapy alone did not yield improved complete remission rates [CR 70.4% (GO group) versus 69.9% (control group), *p* = 0.15]. Overall survival rates, however, were superior in the GO group, although the differences did not reach statistical significance at the final data cutoff (Castaigne et al., 2012). The clinical dilemma of drug sequencing also emerged during several clinical trials in FLT3-mutated AML. For example, in a randomized study of lestaurtinib administered in sequence with intensive chemotherapy for FLT3-mutated AML, the addition of lestaurtinib did not lead to an improved outcome (Levis et al., 2011). Likewise, Knapper et al. (2017) reported that lestaurtinib combined with conventional chemotherapy yielded no overall clinical benefit. The poor outcome in both studies was at least in part due to a substantial and sustained increase in FLT3 ligand (FL) levels in response to chemotherapy. Several studies have convincingly demonstrated that FL, even at low concentrations of 1 ng/ml, leads to autophosphorylation of the FLT3 receptor and thus to a proliferative response in leukemic blasts that cannot be overpowered by the most potent FLT3 inhibitors. Further studies have shown that the surge in FL in response to induction and consolidation chemotherapy becomes more intensive with each subsequent treatment cycle, and that FL levels remain elevated over a course of several weeks (Levis, 2011; Sato et al., 2011). This strongly indicates that FLT3 inhibitors should ideally be administered prior to, or concurrently with, chemotherapy. Such an approach, however, is frequently hampered by the time required to obtain FLT3 mutation status in most centers and the need to start therapy when patients present with high leukemic burden and/or symptomatic disease. As a result, FLT3 inhibitors are typically administered promptly after chemotherapy in most clinical trials (e.g., NCT03250338, NCT03258931, NCT03989713, and NCT02668653). Although targeted agents have shown clinical benefit in the treatment of AML, drug resistance frequently develops over the course of therapy. In a study of chemo-refractory FLT3/ITD-mutated AML, Man et al. (2012) reported that the majority of patients who showed an initial response to sorafenib, as evidenced by clearance or near clearance of BM myeloblasts, lost response after 72 days. Correlative xenograft studies in non-obese diabetic/severe combined immunodeficiency mice transplanted with leukemia cells from patients prior to and after the development of sorafenib resistance identified tyrosine kinase domain mutations at D835 as a mechanism of resistance. Several FLT3-dependent and -independent mechanisms of resistance have been described, ranging from TK point mutations to FL expression and tescalcin-induced intracellular alkalinization (Grundler et al., 2003; Clark et al., 2004; Bagrintseva et al., 2005; Sato et al., 2011; Man et al., 2012). The development of therapeutic resistance to the recently approved IDH inhibitor enasidenib has also been reported. In a letter to *Nature*, Intlekofer et al. (2018) gave account of two IDH2-mutated AML patients who, after achieving a response to enasidenib, developed progressive disease due to emerging second site IDH2 mutations that permitted 2-hydroxyglutarate (2HG) synthesis in the presence of the inhibitor. The authors also reported a similar pattern of resistance in an IDH1-mutated AML patient who developed resistance to the FDA-approved IDH1 inhibitor ivosidenib (Intlekofer et al., 2018). Additional challenges in further paving the way for targeted therapies in AML are represented by the highly complex associations and arrangements of genetic lesions at diagnosis as well as at different time points of the disease. Within this framework, Kottaridis et al. (2002) reported several cases of FLT3 or N-Ras mutations that were detected in relapsed but not in diagnostic samples. Similar findings were reported in the context of whole exome sequencing efforts in paired AML samples by Garg et al. (2015). The genetic diversity in AML was further highlighted by Papaemmanuil et al. (2016) who pinpointed more than 5,000 driver mutations across 76 genes or genomic regions when analyzing genetic and clinical data from 1,540 AML patients. These findings indicate that current risk stratification approaches, which are typically based on the pretreatment assessment of a relatively small panel of genetic markers, might be inaccurate and only provide limited prognostic information, particularly with the advent of targeted inhibitors that have proven beneficial at different disease stages. Indeed, increasing evidence suggests that the prognostic value of current risk stratification models based on the mutational profile of selected genes is contentious due to the extensive molecular heterogeneity of AML. For example, in accordance to current NCCN and ELN risk stratification, the prognosis of NPM1-mutated AML depends on the presence or absence of FLT3/ITD^low/high^. However, recent work by Itzykson et al. (2018) in elderly AML patients strongly suggests that the mutational status of at least five additional genes more accurately defines “risk” and prognosis. In line with these findings, recent data suggest that the response to FLT3 inhibitors depends on the overall mutational profile in FLT3-mutated AML. For example, Tarlock et al. (2019) reported that FLT3/ITD-mutated AML patients with co-occurring NU98-NSD1 fusions, which are more prevalent among the pediatric population, had inferior outcomes compared to other FLT3/ITD-mutated patients, even when treated with a combination of intensive chemotherapy and sorafenib, followed by hematopoietic stem cell transplantation (Jaju et al., 2001). Similarly, it has been established that mutations in WT1 are determinants of poor prognosis, in particular when occurring with FLT3/ITD mutations (Hollink et al., 2009; Tien et al., 2018). These results suggest that even within the subgroup of FLT3-mutated AML, vast heterogeneity—as determined by the co-occurrence of additional mutations—exists which ultimately impacts prognosis and treatment response to targeted inhibitors. As the optimal approach to using currently available FLT3 inhibitors in AML remains to be defined, one potential strategy consists of using the multi-targeted FLT3 inhibitor midostaurin during induction for debulking and adding the more selective FLT3 inhibitor gilteritinib during consolidation with the goal to further reduce and/or eliminate MRD burden. Another predicament is the use of maintenance therapy to reduce the risk of relapse after intensive therapy, including allo-SCT. While previous work has suggested that maintenance therapy after achieving remission is beneficial, most studies were limited by small sample size (Rashidi et al., 2016). As a result, several maintenance trials using immunotherapy, conventional chemotherapy, and/or targeted agents are currently ongoing. Due to stringent eligibility criteria (e.g., patients must have endured induction and consolidation therapies, achieved and maintained a CR, and typically not be a candidate for transplant), enrollment on such trials has been cumbersome.

## Conclusion

The success of imatinib in the management of chronic myeloid leukemia (CML) paved the way for genomics-based, targeted therapies in cancer. After decades of lacking therapeutic progress, an array of targeted agents recently found their way into the clinical AML arena. While most of the recent drug approvals were based on moderate survival benefits for small subgroups of patients, AML generally remains an incurable disease. In fact, allogeneic SCT remains the only chance to achieve long-term disease control or even a cure, which highlights the importance of immunotherapeutic strategies in overcoming drug resistance and disease relapse. Against this background, lines of evidence suggest that immunotherapeutic applications such as checkpoint inhibition, including programmed death 1 (PD-1) or programmed death ligand 1 (PD-L1), may improve the activity of standard chemotherapies and/or targeted agents in AML (Daver et al., 2019). As a result, several clinical studies exploring the antileukemic activity of immune therapy-based combination strategies other than allo-SCT have been developed and are currently under investigation. Although the field appears to generally trend away from traditional chemotherapy for the treatment of AML, it should be noted that combinations of classical “tried-and-trusted” agents such as cytarabine and anthracyclines have the potential to be further exploited. To this end, CPX-351, a fixed, liposomal-encapsulated combination of cytarabine and daunorubicin was approved by the FDA on August 03, 2017, for the treatment of patients with newly diagnosed, therapy-related AML and AML with myelodysplastic changes. The approval was based on a significant survival benefit of 9.56 months in the Vyxeos versus 5.95 months in the standard chemotherapy arm in a trial of 309 AML patients (Lancet et al., 2018). Ongoing studies are exploring other investigational agents and combination concepts. It is to be expected that the recently amassed amount of knowledge in AML will continue to translate into novel treatment approaches that are vastly different from traditional chemotherapies and further improve outcomes. These achievements, however, must be in sympathy with advances in the development of companion diagnostics and the design of innovative clinical trials to make effective therapies available to the patient sooner.

## Author Contributions

Both authors listed have made a substantial, direct and intellectual contribution to the work, and approved it for publication.

## Conflict of Interest

The authors declare that the research was conducted in the absence of any commercial or financial relationships that could be construed as a potential conflict of interest.
